# Editorial: Kawasaki disease: an ongoing enigma tangled with the appearance of MIS-C

**DOI:** 10.3389/fped.2023.1209723

**Published:** 2023-05-30

**Authors:** Andras Bratincsak, Marianna Fabi

**Affiliations:** ^1^ Department of Pediatrics, John. A Burns School of Medicine, University of Hawaii, Honolulu, HI, United States; ^2^ Department of Pediatrics, University of Bologna, Bologna, Italy

**Keywords:** Kawasaki disease, MIS-C multisystem inflammatory syndrome in children, coronary artery dilation, COVID-19, differential diagnosis

**Editorial on the Research Topic** Kawasaki disease: an ongoing enigma tangled with the appearance of MIS-C

Kawasaki disease (KD) continues to present a diagnostic and therapeutic conundrum for pediatrics. More than 50 years after the first publication by Dr. Tomisaku Kawasaki (1), and decades of discovery and advancement in the diagnosis and treatment of KD, the etiology of the most common acquired heart disease in children is still not known. In the past few years, the story of KD got tangled with a new disease entity: COVID-19. The first human SARS-CoV-2 infection was recognized in the Wuhan province of China in late 2019 (2), and spread around the globe in 2020. The COVID-19 epidemic has become the largest pandemic in modern history, similar to the Spanish flu in 1918–1920. COVID changed our entire world, limited our social interactions, altered the health care, and challenged our trust and dedication in our medical profession. COVID also changed how we take care of our patients with KD.

In early 2020, a novel disease entity was recognized in Italy and Great Britain, presenting a few weeks after acute COVID-19 infection. Pediatricians noticed a constellation of clinical features resembling KD, presenting with fever, conjunctival injection, adenopathy and cardiac involvement in children and adolescents (3, 4). The cardiac involvement included dilation of coronary arteries and decreased left ventricular systolic function, presenting as a mixture of atypical KD and myocarditis. This new disease, first called Pediatric Inflammatory Multisystem Syndrome (PIMS), later named Multisystem Inflammatory Syndrome in Children (MIS-C), overlapped with the diagnostic criteria of KD and created a significant dilemma for differential diagnosis (5). Fortunately, steroid and IVIG administion within a few days of diagnosis effectively treated the new entity in the vast majority of cases. Although there is an overlap in the clinical presentation and the treatment of KD and MIS-C, there are important differences bearing diagnostic and therapeutic implications affecting the morbidity and mortality of these children.

In this special issue, we focused on how the diagnosis and management of KD evolved in the era of a novel and similar disease: MIS-C. We gained important insights of the epidemiology of both KD and MIS-C. Kim et al. conducted one of the largest nationwide retrospective studies of KD in Korea, and concluded that besides a still unknown infectious agent, important genetic and environmental factors must play a role in KD. On the other hand, Semeraro et al. demonstrated how the severity of acute COVID-19 infection and the prevalence of MIS-C among hospitalized children changed according to the SARS-CoV-2 variants.

Universal mask-wearing in the beginning of the pandemic resulted in a dramatic decrease in the prevalence and slight change in the age-distribution of KD. The diagnostic dilemma of KD and MIS-C, especially in ages 6 and older, provoked the question whether KD presentation might have changed after COVID-19 infection due to a different immune response. Besides realizing the different prevalence of MIS-C and KD among various ethnic groups, You et al. demonstrated that certain laboratory markers, such as albumin, may aid in discerning between the two entities. MIS-C and KD do present with different clinical features, however most of the clinical findings are very similar, and the increasing number of positive COVID serologies among children created an even harder task for pediatricians to correctly diagnose patients.

This special issue also contains further analysis of promising new diagnostic markers for KD. Zhong et al. showed that circulating non-coding RNAs, mostly microRNA can be not only diagnostic for acute KD compared to other febrile illnesses, but may differentiate acute and convalescent KD as well as KD with and without coronary artery involvement. Mauro et al. presented a comprehensive review of neurological symptoms and conditions in KD and MIS-C, outlining important similarities and differences, and suggesting the importance of long-term follow-up for appropriate management of neurological sequelae. Another relevant aspect of KD and MIS-C was summarized in the review by Jose and Tierney, reflecting the range of coronary artery involvement and cardiac dysfunction noted in patients with MIS-C, emphasizing the importance of creating a standardized follow-up schedule for this disease entity, similar to what we have for KD.

COVID is here to stay, and so is MIS-C, creating an ongoing differential diagnostic problem for clinicians to provide optimal management. Several authors concluded in this issue that novel diagnostic modalities are needed to enhance the differential diagnosis of the two conditions. A powerful diagnostic test would help differentiate borderline cases, late presentations, incomplete or atypical KD, and MIS-C occurring in younger age groups. A proper diagnostic test or biomarker would accurately distinguish the two conditions and would be available for practical clinical use. A powerful diagnostic biomarker, as suggested in a previous special issue on KD in Frontiers in Pediatrics (6), would be able to identify patients at risk of developing cardiac complications, such as coronary aneurysms or left ventricular dysfunction.

In the future, basic science research, translational and clinical studies in KD and MIS-C should concentrate on:

1. finding a sensitive and specific test or algorithm distinguishing KD and MIS-C
2. identifying children at risk for cardiac complications during initial diagnosis
3. establishing long-term follow-up guidelines for MIS-C, and
4. evaluating specific therapies to decrease coronary artery aneurysm in both conditions.
